# Core self-evaluations and work engagement: Testing a perception, action, and development path

**DOI:** 10.1371/journal.pone.0182745

**Published:** 2017-08-07

**Authors:** Maria Tims, Jos Akkermans

**Affiliations:** Department of Management and Organization, Faculty of Economics and Business Administration, Vrije Universiteit Amsterdam, Amsterdam, The Netherlands; IUMPA—Universitat Politecnica de Valencia, SPAIN

## Abstract

Core self-evaluations (CSE) have predictive value for important work outcomes such as job satisfaction and job performance. However, little is known about the mechanisms that may explain these relationships. The purpose of the present study is to contribute to CSE theory by proposing and subsequently providing a first test of theoretically relevant mediating paths through which CSE may be related to work engagement. Based on approach/avoidance motivation and Job Demands-Resources theory, we examined a perception (via job characteristics), action (via job crafting), and development path (via career competencies). Two independent samples were obtained from employees working in Germany and The Netherlands (*N* = 303 and *N* = 404, respectively). When taking all mediators into account, results showed that the perception path represented by autonomy and social support played a minor role in the relationship between CSE and work engagement. Specifically, autonomy did not function as a mediator in both samples while social support played a marginally significant role in the CSE–work engagement relationship in sample 1 and received full support in sample 2. The action path exemplified by job crafting mediated the relationship between CSE and work engagement in both samples. Finally, the development path operationalized with career competencies mediated the relationship between CSE and work engagement in sample 1. The study presents evidence for an action and development path over and above the often tested perception path to explain how CSE is related to work engagement. This is one of the first studies to propose and show that CSE not only influences perceptions but also triggers employee actions and developmental strategies that relate to work engagement.

## Introduction

The current labor market emphasizes individual self-management and proactive behaviors, increasing the need for employees to feel capable, competent, and in control of their work environment to uphold and develop themselves. A personality construct that closely resembles these fundamental assessments of one’s self-worth, is core self‐evaluations (CSE; [1]). CSE is a broad personality construct, consisting of self-esteem, generalized self-efficacy, emotional stability, and locus of control [2]. CSE theory suggests that the evaluation of self-worth and capabilities is highly relevant for how employees perceive their work and how they behave at work. High levels of CSE are, for example, positively related to job satisfaction and performance [3]. A recent review of the CSE literature [4] concluded that important information is lacking about the role of CSE in explaining work outcomes as most researchers have not examined *why* CSE relates to outcomes. Another conclusion of the review was that earlier studies almost exclusively focused on job satisfaction and performance while ignoring other important outcomes. Heeding both of these calls, the present study aims to: (1) contribute to a better understanding of why CSE relates to work outcomes by examining several proposed mediators concurrently, and (2) to extend the current CSE findings to other outcomes (i.e., work engagement). To do so, we use the approach/avoidance framework [5] to suggest mediators and Job Demands-Resources theory [6] to integrate the different mediational paths and explain the relationship between CSE and work outcomes.

More specifically, related to the question why CSE is related to outcomes, the studies that have examined mediators thus far have mostly argued that people with high CSE are more likely to *perceive* their job characteristics positively, which, in turn, would lead to a positive perception of the job (i.e., a “perception path”) [7]. Because of the approach motivation (i.e., behavior driven by or directed toward positive stimuli) [5] that is associated with CSE [8], high CSE individuals are more likely to view their experiences at work positively and are less likely to focus on negative information [4, 9].

Judge and colleagues [1, 2] also proposed another mechanism through which CSE may be related to work outcomes, namely by influencing the actions of individuals [4], which we call an “action path”. Several studies have aimed to examine the action path by assessing motivation or goal persistence [3, 10] as mediators because employees with high CSEs are expected to be more motivated to perform well. However, it is debatable whether the action path referring to the actions people take is best represented by constructs such as motivation, because motivation is an evaluation that may trigger action, but it is not an action in itself. We propose that job crafting may be a representative of the “action path”. Job crafting, positioned in the JD-R model, can be defined as “the changes that employees make to balance their job demands and job resources with their personal abilities and needs” [11, p. 147]. Considering that CSE is a positive emotional construct that encourages specific action tendencies [12], individuals with high CSEs may be more likely to take proactive action at work [13]. Job crafting, in turn, has been found to be associated with job satisfaction and work engagement [11]. Thus, it is worthwhile to examine whether job crafting could act as an “action” mechanism in the relationship between CSE and work engagement.

Furthermore, we also propose a third path, namely a “development path”, in which people with high CSEs are expected to be more likely to develop career-related competencies, and subsequently experience better outcomes. We propose this additional path because individuals with high CSEs are also more likely to positively appraise their career competencies, which reflects a career-related personal resource [14, 15]. According to JD-R theory, personal resources refer to a sense of being able to control and impact the environment [16]. Career competencies refer to the knowledge, skills, and abilities central to career development [17], such as reflecting on one’s motivation and identity, skills and expertise, and relationships and reputation [18]. In line with Johnson et al. [13], we expect that CSE not only color perceptions (i.e., “perception path”) and initiate action (i.e., “action path”), but also make people more likely to engage in self-regulation in their career, which is necessary to create opportunities for themselves and their organization [19].

**Fig 1:**
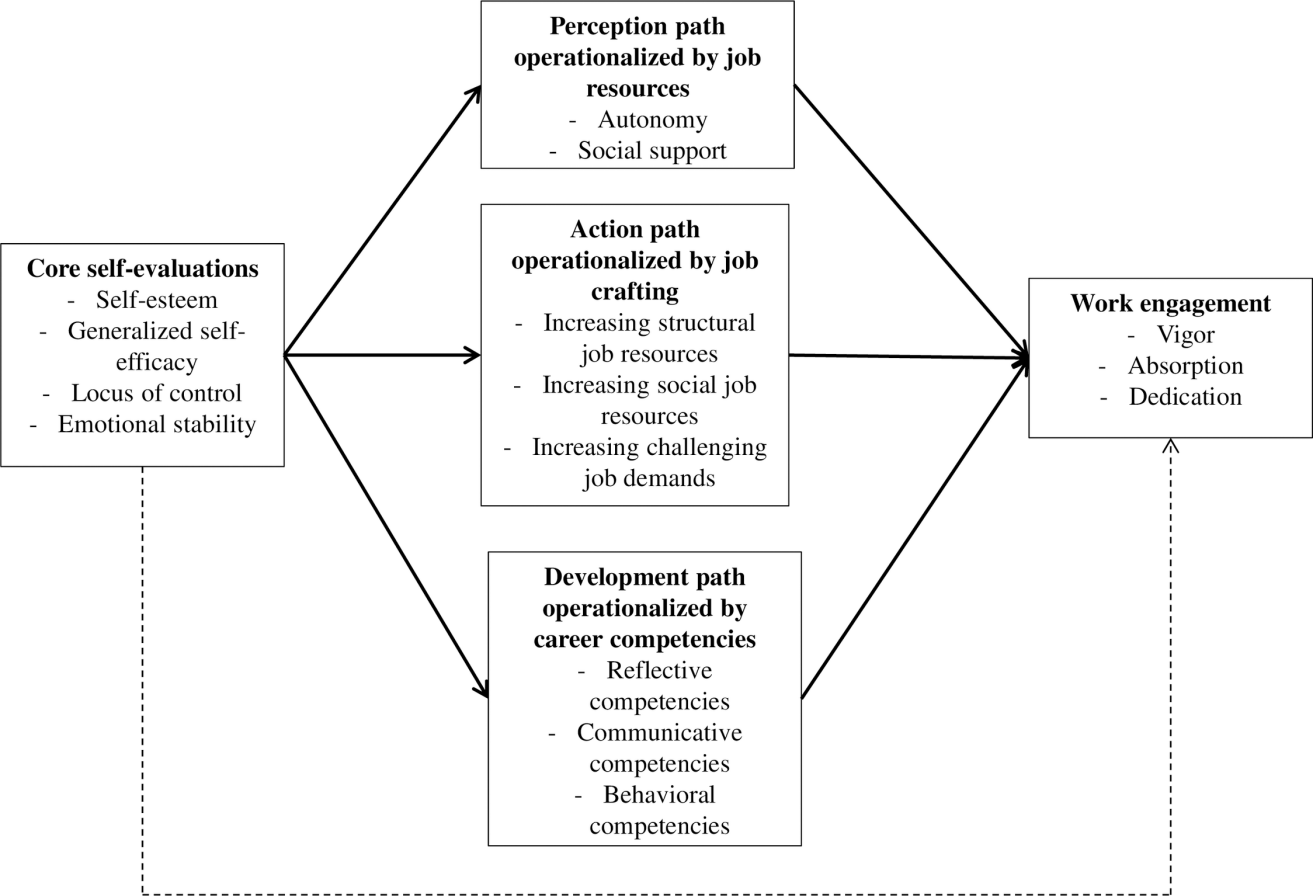
Conceptual model showing the relationship between core self-evaluations and work engagement mediated by the perception, action, and development path. The solid lines indicate the indirect paths while the dashed line represents the direct relationship between core self-evaluations and work engagement.

By integrating these three paths using approach motivation and JD-R theory, we aim to contribute to the CSE literature by examining whether CSEs form the basis on which people *perceive* their job, *craft* their job, and *develop* their career, which, in turn, may relate to work outcomes. Building on previous studies that mainly focused on one type of mediator (i.e., the “perception path”), we provide a first empirical test of the three mediators concurrently (sample 1). This is important because this research highlights that core self-evaluations influence cognitive, behavioral, and developmental processes that are needed to fuel important work outcomes. Testing these differential paths simultaneously, allows us to examine the strength of each path in comparison with the other paths. We also contribute by extending the current CSE findings to other outcome measures [4] as we focus on work engagement [20]. The research model is displayed in Fig 1. Moreover, to lend further credibility to these initial findings, we examined the proposed research model in a second sample from a different country (sample 2). This design allows us to test whether the findings in one particular sample can be cross-validated in a second sample in a different context.

### CSEs and work engagement

CSE is defined by four higher-order concepts, namely self-esteem, generalized self-efficacy, locus of control, and emotional stability [21]. Self-esteem refers to how people evaluate their own self-worth, and generalized self-efficacy captures whether individuals trust to have the ability to perform and cope successfully across life situations [1]. Locus of control refers to one’s belief to be able to impact the environment to reach desired outcomes, and finally, emotional stability reflects the disposition to feel calm and secure and a sensitivity to positive emotional states [8]. Together, these individual traits increase the prediction of work outcomes because they reflect a broad measure of how individuals see themselves. As such, CSE is argued to be more than just an aspect of an individual’s self-worth [22, 23].

CSE has been proposed as a means to understand the influence of dispositional tendencies on how individuals perceive their work [9]. That is, the way individuals evaluate themselves forms the basis on which they evaluate other, more specific aspects of work, such as job satisfaction [1, 24] and work engagement [10]. Whereas many studies have shown a positive relationship between CSE and job satisfaction, the present study focuses on a more active evaluation of the job, namely work engagement. Work engagement is defined as a “positive work-related state of fulfillment that is characterized by vigor, dedication, and absorption” [25, p. 701]. Vigor refers to high levels of energy, dedication refers to enthusiasm and involvement in one’s work, and absorption arises when one is fully concentrated at work in such a way that it is difficult to detach oneself from work [25]. Given that positive CSEs make employees more likely to focus on the positive, stimulating, and challenging aspects of their work [1], we expect that CSE relates positively to work engagement. Furthermore, given that positive self-evaluations seem to trigger an approach motivation toward positive outcomes and minimize an avoidance motivation to prevent negative outcomes [8], high-CSE individuals should try to establish positive outcomes. Indeed, work engagement as a positive work-related state is likely to result from an approach motivation [20], and Rich et al. [10] reported a positive relationship between CSE and work engagement.

Hypothesis 1: CSE is positively related to work engagement

Judge and colleagues [2] suggested that CSE can influence outcomes directly via spillover of positive self-views on outcomes; or indirectly via cognitions and appraisals (i.e., perceptions) or via actions of the individual. Furthermore, CSE could influence work outcomes as a moderator. After reviewing the literature on mediation models, Chang et al. [4] concluded that studies had mostly focused on the direct relationship between CSE and outcomes or examined the “perception path”. In the present study, we aim to develop a better understanding of the proposed mediational paths that link CSE to work outcomes. Thus, we don’t focus on one particular indirect mechanism but examine them simultaneously. More specifically, we focus on (1) a direct path between CSE and work engagement (as discussed above); (2) an indirect path via perceptions; (3) an indirect path via actions; and (4) an indirect path via career development.

### The perception path

Perceptions of job characteristics are typically found to be higher among individuals with positive CSEs [23] because they are likely to seek out situations in which positive conclusions can be derived [26]. For example, Stumpp et al. [7] included the job characteristics autonomy, skill variety, task identity, task significance, and feedback from the job as mediators of the relationship between CSE and job satisfaction. Results showed that CSE related positively to all job characteristics, indicating that those with higher CSEs rated their job characteristics more positively compared to those with lower CSEs.

The present study seeks to first replicate these results from previous studies by examining the relationship between CSE and work engagement via perceived job characteristics. In line with JD-R theory [6], we focus on job resources in this study. Job resources refer to work aspects that enable employees to do their work (e.g., autonomy) and that can buffer the effects of certain stressors (e.g., social support). Because of the motivating potential of job resources, they have often been found to relate positively to work engagement [27]. Particularly autonomy and social support are strongly related to one’s evaluation of the job. To explain, autonomy creates the perception that the environment is controllable by being able to make certain choices in work, and social support makes the work environment more pleasant and rewarding [28]. In turn, these feelings arouse work engagement. Based on CSE theory–which states that CSE affects thinking processes and specific appraisals [2]–and based on JD-R theory–which states that the availability of job resources in the work environment leads to work engagement–we expect that individuals with higher levels of CSEs will evaluate their job resources more positively [1, 23], which, in turn, will be related to work engagement [29]. As a practical example, a teacher with high CSEs is likely to have a more positive outlook on the job resources in his job–for example recognizing the autonomy of designing classes and the support provided in the teacher room during breaks–and subsequently becomes more engaged at work.

Hypothesis 2: Perceptions of job resources (i.e., autonomy and social support) mediate the relationship between CSE and work engagement

### The action path

Another mechanism through which CSE may relate to work engagement, is the action path. The action path implies that CSEs guide an individual’s actions. To achieve desired outcomes, employees can use proactive behaviors [30] to change work environments or themselves [31]. When employees proactively change their work environment, this is called job crafting [32]. Recently, job crafting was placed within JD-R theory to study job crafting from the job (re)design perspective [33]. In this perspective, job crafting refers to making changes in job resources and job demands. Employees craft their job resources by increasing them because of their motivating potential [34]. Furthermore, job demands can be crafted either by decreasing those aspects that hinder achieving work goals, or by increasing those job demands that are considered challenging though still require effort [35]. Tims et al. [11] distinguished four job crafting dimensions: (1) increasing structural job resources (i.e., mobilizing autonomy, variety, and opportunities for development), (2) increasing social job resources (i.e., mobilizing social support, coaching, and feedback), (3) increasing challenging job demands (i.e., mobilizing working on new projects), and (4) decreasing hindering job demands (i.e., cognitive and emotional job demands). We focus on the first three job crafting dimensions–sometimes referred to as “expansive job crafting”–because CSEs represent a positive outlook. The decreasing hindering job demands would be less fitting here as it has been found to be associated with less work engagement, more burnout, and lower job performance [11, 36, 37].

Employees who hold certain expectations about their job will prioritize certain job characteristics over others [38]. By crafting their job, employees create a work environment that fits their preferences, skills, and abilities, thereby fueling work engagement [33, 37]. Employees with a positive self-view are more likely to create a job that facilitates work engagement. Specifically, employees with high CSEs believe that they are capable to ensure that their actions will bring desired outcomes (i.e., high self-efficacy and internal locus of control), thus enabling them to independently take action at work. When employees are confident that they can influence their environment, they may be more likely to actually make changes [39], as was evidenced by Tims et al. [36], who found a positive relationship between self-efficacy and job crafting. Similarly, employees with an internal locus of control believe that they can determine outcomes with their behavior and are therefore more likely to take action [40]. The approach motivation associated with CSE may also elucidate why employees with high CSEs may be more inclined to proactively shape their job characteristics through job crafting: approach motivation drives individuals to attain positive outcomes which oftentimes involves self-initiated behaviors to obtain something positive that is missing or to maintain something positive that is already present [5]. Applied to the work environment, work engagement may be obtained or maintained by proactively changing aspects of work. In sum, in addition to a positive perception of job characteristics (i.e., the aforementioned perception path), high CSEs may also increase job crafting behaviors that actively change those characteristics (i.e., an action path).

Job crafting, in turn, has been associated with work engagement because individuals craft their job in such a way that they create a resourceful and energetic work environment [41]. Following JD-R theory [6], a job in which job resources are available and employees feel challenged, will contribute to work engagement because employees have the means available to deal with their work tasks. By increasing job resources and introducing challenging tasks the job is likely to better fit the employee and enhance the meaning of work [42]. Thus, we argue that individuals with high CSEs are likely to engage in expansive job crafting behaviors, which would subsequently be associated with higher levels of work engagement. As an example, a consultant who is self-efficacious and has high self-esteem would be more likely to proactively generate resources–for example creating more autonomy by combining several customers on one day which allows her to work from home the next day. This proactive arrangement would make her more engaged in her work as it fits her scheduling preference.

Hypothesis 3: Job crafting actions mediate the relationship between CSE and work engagement

### The development path

Whereas the perception and action paths had already been discussed in previous research [4], we next propose a new path through which CSE may be related to work engagement, which is based on findings that CSEs also seem to play a role in career studies. For example, Judge and Hurst [43] examined the relationship between CSEs and career success trajectories. Their study showed that those individuals who hold positive CSEs at the start of their careers, start in better jobs and advance quicker in their career compared to those with lower initial levels of CSEs. Furthermore, career development processes have been shown to relate positively to work engagement and to act as a mediator between personal variables and work-related outcomes [14, 15]. Hence, we argue that CSE would also relate to work engagement via a career development path. Building on this, we focus on career competencies because CSE literature suggests that individuals with positive CSEs may be more likely to engage in self-initiated career planning, exploration, and job search [19], which are key characteristics of career competencies. Indeed, CSEs may trigger employees’ self-awareness [44], which can be used to acquire knowledge about important career skills that need to be developed [38]. Furthermore, the positive emotions associated with CSEs are important antecedents of the development of specific career-related skills and competencies [45].

Current operationalizations of career competencies indicate that they consist of three interrelated competencies [17]: reflective, communicative, and behavioral career competencies. Reflective career competencies refer to a person’s reflection on career motivation (e.g., what are my values, passions, and motivations in my career?) and qualities (e.g., what are my strengths, shortcomings, and skills for my career?). Communicative career competencies deal with networking (e.g., whom do I know that are of value for my career?) and self-profiling (e.g., to whom and how should I present my knowledge, skills, and abilities?). Finally, behavioral career competencies refer to work exploration (e.g., how can I find opportunities for further education?) and career control (e.g., what are my long-term goals and how can I plan on attaining them?).

Based on approach motivation, it is likely that higher CSEs would be associated with higher levels of career competencies as these positive self-evaluations should result in individuals having confidence in themselves to reach positive and desired career-related goals. To explain, CSE is argued to lead to a preference and action toward more challenging and complex work, a feeling of control over life events and thus also over career-related events [46], and career-related planning [19]. Hence, employees with positive CSEs should be more likely to master career competencies that enable them to actually take control over their work and career.

Some empirical results support our reasoning. For example, Akkermans et al. [17, 47] report a positive relationship between career competencies and generalized self-efficacy, which is one of the four CSE traits. This means that individuals who positively evaluate their general competencies (i.e., across different contexts) were more likely to develop their career competencies. Similarly, King [30] found that self-efficacy and desire for control were predictors of career self-management, and Dougherty, Cheung, and Florea [48] proposed that employees with high CSEs are more likely to approach others and to form networks that help them to develop themselves in a way that is consistent with their goals. Evidence from these separate studies suggests that employees who positively evaluate themselves, are likely to develop and possess career competencies.

In turn, based on JD-R theory, the relationship between career competencies and work engagement can be explained by the motivating potential of personal resources [49]. Personal resources refer to feeling able to control and impact the environment [16]. Research has shown that career competencies function similar to personal resources and were related to higher levels of work engagement [14]. Individuals who believe they possess sufficient career-related competencies are more likely to feel energized and engaged. Hence, we expect that career competencies will mediate the positive relationship between CSE and work engagement, which we refer to as the “development path” of CSE.

As a practical example, a police officer who believes she has control over her own career path (i.e., high locus of control) would be more likely to actively think about potential career paths within–or outside of–the police force, and start setting goals for herself. Reflecting on her passions and strengths, and actively pursuing career goals would then provide her with a clear direction and with renewed energy, likely leading to an increase in her engagement at work.

Hypothesis 4: Career development competencies mediate the relationship between CSE and work engagement

## Method and results: Sample 1

### Procedure and participants

Data were collected among a heterogeneous group of German employees working in different sectors and occupations. Participants were recruited via the social networks (e.g., LinkedIn) of the researchers and student assistants. Only employees aged between 16 and 30 years could participate in this study because this research was part of a research project among young employees. CSE is likely developed already in young age as it represents a broad personality trait and has been measured among student samples [3]. Job characteristics, proactive behavior, and the development of career competencies are relevant for employees of all ages, but particularly for younger employees [17], making this data suitable to examine the proposed model. Employee reports were used to collect data because it has been argued that employees are the ones who are best able to judge their personality and constructs such as work engagement [50] and because we are interested in perceived job characteristics and career competencies [23]. Furthermore, job crafting is often not visible for peers or supervisors [32], thus making it difficult for others to assess the job crafting behaviors of colleagues.

Prior to conducting the study, the University Research Ethics Review Checklist indicated that a research ethics review was not necessary. Reasons for this decision are that the study involves no manipulations, no children, and participants were assured that their data is treated anonymously. Furthermore, all participants provided informed consent by clicking on the link to start the research and were informed that they could withdraw from the study at any time.

Power analysis using G*Power version 3.0.10 [51] indicated that for 80% power to detect a small effect size when the correlation in the population is .25 (based on the meta-analysis of Judge & Bono [52]), a sample of 259 participants is needed. Our sample consisted of 303 participants (63.4% female). Mean age of the respondents was 24.2 years (SD = 3.7). Participants worked on average 34.7 hours a week (SD = 17.3) and were tenured in their current organization on average for 2.0 years (SD = 1.8). Total years of work experience was on average 3.3 years (SD = 3.0). Educational level of the respondents was relatively high with 38.9% of the respondents who finished higher vocational education, 26.1% finished university, and 15% finished intermediate vocational education. Respondents worked in the following occupational groups (ESCO classification): service and sales workers (40.3%), professionals (38.2%), plant and machine operators and assemblers (10.6%).

### Measurement instruments

Core self-evaluations were measured with the 12-item Core Self-Evaluations Scale (CSES) developed by Judge et al. [21]. An example item is “I am confident I get the success I deserve in life.” Response categories ranged from 1 (*strongly disagree*) to 5 (*strongly agree*). Reliability estimates of all scales are presented in Table 1 (all > .70).

**Table 1:** Correlation table: Study 1 (*N* = 303) correlations are presented below the diagonal / Study 2 (*N* = 404) correlations are presented above diagonal.

	1	2	3	4	5	6	7	8	9	10
1 Core self-evaluations	.84/.82	.33**	.23**	.37**	.14**	.26**	.35**	.48**	.40**	.45**
2 Autonomy	.22**	.85/.88	.15**	.44**	.06	.31**	.20**	.22**	.09	.32**
3 Social support	.26**	.21**	.90/.92	.37**	.58**	.26**	.18**	.34**	.20**	.43**
4 Increasing struct. JR	.36**	.35**	.30**	.77/.79	.50**	.66**	.39**	.39**	.37**	.51**
5 Increasing soc. JR	.12*	.05	.41**	.42**	.75/.82	.51**	.22**	.40**	.28**	.40**
6 Increasing chall. JD	.23**	.25**	.16**	.66**	.47**	.77/.80	.31**	.34**	.25**	.41**
7 Refl. competencies	.49**	.23**	.17**	.39**	.18**	.43**	.80/.83	.49**	.41**	.37**
8 Comm. competencies	.51**	.21**	.30**	41**	.34**	.39**	.56**	.81/.79	.46**	.47**
9 Behav. competencies	.51**	.20**	.15**	.38**	.12*	.33**	.56**	.61**	.86/.82	.30**
10 Work engagement	.38**	.35**	.33**	.56**	.40**	.50**	.56**	.48**	.41**	.92/.95

Note. Cronbach’s alphas are presented on the diagonal (Study 1/Study 2). Struct. = structural; JR = job resources; soc. = social; chall. = challenging; JD = job demands; Refl. = reflective; Comm. = communicative; Behav. = behavioral.

** *p* < .01.

* *p* < .05.

Job resources were measured with two short scales developed by Bakker, Demerouti, Taris, Schaufeli, and Schreurs [53]. Autonomy was measured with three items (e.g., “Can you decide on your own how your work is executed?”). Supervisory social support was measured with three items (e.g., “If necessary, my supervisor helps me with a certain task”). Both response categories ranged from 1 (*never*) to 5 (*always*).

Job crafting was assessed with the Job Crafting Scale [11]. We used 15 items to assess the three dimensions of expansive job crafting. Increasing structural job resources was measured with five items (e.g., “I make sure that I use my capacities to the fullest”), increasing social job resources was measured with five items (“I ask whether my supervisor is satisfied with my work”), and increasing challenging job demands was also assessed with five items (e.g., “When an interesting project comes along, I offer myself proactively as project co-worker”). Response categories ranged from 1 (*never*) to 5 (*very often*).

Career competencies were measured with the Career Competencies Questionnaire [17]. The scale uses 21 items to assess the three underlying dimensions (7 items for each dimension): Reflective career competencies (e.g., “I can clearly see what my passions are in my work”), communicative career competencies (e.g., “I can clearly show others what my strengths are in my work”), and behavioral career competencies (e.g., “I can make clear career plans”). Response categories ranged from 1 (*strongly disagree*) to 5 (*strongly agree*).

Work engagement was measured with the 9-item version of the Utrecht Work Engagement Scale [25]. The scale contains three items to assess vigor (e.g., “At my work, I feel bursting with energy”), dedication (e.g., “I am enthusiastic about my job”), and absorption (e.g., “I get carried away when I am working”). A seven point response scale with answering categories ranging from 0 (*never*) to 6 (*always*) was used.

### Analysis strategy

Structural equation modeling (SEM) using AMOS 22 [54] was used to test the hypothesized model. Before performing the SEM analyses, confirmatory factor analyses (CFA) were performed to test the factor structure of the scales. A CFA using latent variables was performed for each scale and based on these results the fit of the measurement model was examined. To determine model fit, we used the χ^2^ goodness-of-fit statistic and the Root Mean Square Error of Approximation (RMSEA). In addition, the Comparative Fit Index (CFI) and the Tucker-Lewis Index (TLI) were inspected. Values of .90 and higher for CFI and TLI while values lower than .08 are considered as indicating adequate fit [55]. Because several mediators were tested simultaneously, we used phantom models to obtain estimates and tests of the specific effect of each mediator [56] in the relationship between CSE and work engagement. A phantom model is necessary to receive estimates of each of the indirect effects separately, rather than one estimate for the indirect effect that takes into account all mediators simultaneously. A phantom model consists of latent variables that represent the variables involved in the specific effect. For example, three latent phantom variables are added to the SEM model when testing the specific effect of CSE on work engagement via autonomy. The parameters of the latent phantom variables are constrained to the values of the paths in the SEM model, thereby not influencing the estimation of the SEM model [56]. The phantom model has no meaning but allows the program to provide estimates of the specific effects.

### Confirmatory factor analyses

The CFA consisted of six latent variables with the items or scale means as indicators (i.e., CSE (12 items), autonomy (3 items), social support (3 items), job crafting (3 dimensions), career competencies (3 dimensions), and work engagement (3 dimensions)). Table 2 presents the results of the CFA. Fit of the initial measurement model was insufficient. Fit was improved by using modification indices and specifying correlations between items that belonged to the same scale or between items from different scales but with similar meaning. First, five items of the CSES were correlated with each other. The CSES was developed to capture commonality among the four CSE traits [21], which means that items may reflect a combination of different CSE traits. This commonality likely resulted in some shared variance in items of the CSES. Second, a correlation between an autonomy and supervisor support item with similar content needed to be specified, and between increasing social job resources (job crafting) and social support. Fit indices then reached acceptable levels (see Table 2). However, before proceeding with this model, we examined two additional CFAs in which autonomy and supervisor support; and increasing social job resources and social support were modeled as one factor. Model fit was clearly unacceptable in both cases (χ^2^ = 641.89, *df* = 14, CFI = .47, TLI = .21, RMSEA = .39, and χ^2^ = 243.27, *df* = 9, CFI = .74, TLI = .57, RMSEA = .29) and we therefore conclude that the constructs are in fact distinct even though one item from the respective scales was allowed to correlate. Factor loadings of the measurement model ranged between .39 (CSE) and .94 (supervisor support; all *p*’s < .001).

**Table 2:** Results of confirmatory factor analyses (CFA).

	Sample 1 (*N* = 303)					
	χ^2^	*df*	CFI	TLI	RMSEA	∆χ^2^/∆df
CFA	765.42**	334	.89	.88	.07	
CFA trimmed	678.44**	328	.91	.90	.06	86.98/6**
CFA competing	1773.34**	341	.63	.59	.12	1094.90/13**
Structural model	676.20**	328	.91	.90	.06	
			Sample 2 (*N* = 404)			
CFA	998.80**	335	.88	.86	.07	
CFA trimmed	685.91**	325	.93	.92	.05	312.89/10**
CFA competing	1994.20	338	.69	.66	.11	1308.29/13**
Structural model	773.48**	326	.92	.90	.06	

** *p* < .01.

Additionally, the fit of the hypothesized measurement model was compared to the fit of a competing measurement model. The competing CFA model examined the fit of a model in which autonomy and increasing structural job resources; social support and increasing social job resources; and CSE and career competencies each loaded on the same factor to examine whether perceptions of job characteristics can be statistically distinguished from job crafting behaviors, and whether CSE is different from more specific career competencies. Work engagement was modeled as a separate latent factor. As evident from Table 2, fit of this model was significantly worse compared to the hypothesized model. For the six latent variables measured with multiple indicators, the composite reliabilities and average variance extracted (AVE) estimates were as follows: CSE (.84, .31), autonomy (.85, .59), social support (.89, .59), job crafting (.77, .54), career competencies (.80, .58), and work engagement (.89, .73). In line with the competing CFA, these values also suggest discriminant validity, in that the square root of the AVE for a specific construct was greater than the factor correlations between that construct and other constructs in the study [57]. This applied to all study variables except for the correlation between CSE and career competencies, which was .69. Although these constructs share some theoretical basis, neither the competing CFA indicated that they were similar, nor is the correlation higher than .80 [58].

We proceeded with the analysis of CMV by adding an orthogonal factor to the measurement model [59, 60]. The orthogonal factor was modeled with paths to all 27 indicators of the six latent variables in the model. The method factor improved model fit (χ^2^ = 570,79, *df* = 302, Δχ^2^ = 136,55/Δ*df* = 28, *p* < .01, CFI = .93, RMSEA = .05), indicating that unequal method effects are present. The median amount of method variance in the 27 indicators was 20% (for comparison, Williams & McGonagle [61] report a similar value for their test of an orthogonal method factor of 17.2%; both values are lower than Williams, Cote, and Buckley [62], who reported a median amount of method variance of 25% in prior studies. Furthermore, an orthogonal method factor represents a conservative test of the relationships [61], thus, possibly overestimating CMV.

### Hypotheses testing

In Hypothesis 1, we expected a positive relationship between CSE and work engagement. When all other structural paths in the model were constrained to zero, results showed that the relationship between CSE and work engagement was significant (*β* = .46, *p* < .01). While testing mediation hypotheses 2 through 4, all paths were included to examine which of the mediational paths represents the strongest mechanism between CSE and outcomes. In line with Hypothesis 2, results showed that CSE positively related to the job resources autonomy (*β* = .25, *p* < .01) and social support (*β* = .22, *p* < .01). However, autonomy and social support were not related to work engagement (*β* = .10, *p* = .064; *β* = .09, *p* = .077, respectively). Together, these results reject Hypothesis 2. When testing the specific mediation of autonomy and social support using phantom models, the results showed that the specific effects via autonomy and social support were marginally significant (respectively, estimate = .05, *SE* = .03, BCC: .006–.109, *p* = .056; estimate = .04, *SE* = .029, BCC: .004–.098, *p* = .067). Thus, only when examining these specific relationships, there is an indication that these job resources may play a role in the CSE–work engagement relationship. However, when including the other two mediators (i.e., job crafting and career competencies), both job resources are not related to work engagement anymore.

In line with Hypothesis 3, results showed that CSE related positively to job crafting (*β* = .41, *p* < .01). In turn, job crafting was positively related to work engagement (*β* = .38, *p* < .01). Using the phantom model approach, the estimate for this specific effect was found to be significant: estimate = .31, *SE* = .10, BCC: .175–.495, *p* = .001. These results support Hypothesis 3.

Testing hypothesis 4, we found that CSE related positively to career competencies (*β* = .71, *p* < .01), which, in turn, related to work engagement (*β* = .44, *p* < .01). The estimate of this specific effect obtained via a phantom model was .61, *SE* = .22, BCC: .328–1.028, *p* = .001. These results support the mediational role of career competencies in the relationship between CSE and work engagement (Hypothesis 4).

The direct relationship between CSE and work engagement disappeared when the model included the mediators (from *β* = .46, *p* < .01 to *β* = -.06, *p* = .499), indicating that both job crafting and career competencies mediated the relationship between CSE and work engagement, providing support for the action and development paths. Because this is the first study to test these three mediational paths simultaneously, we cross-validated our findings in a new sample in a new context before interpreting the results.

## Method and results: Sample 2

### Procedure and participants

The procedure for data collection was similar to the procedure of sample 1 albeit that this data was collected in The Netherlands. The sample consisted of 403 participants (40.4% female). Mean age of the participants was 26.1 years (SD = 5.0). On average participants worked 33.6 hours a week (SD = 14.4) and were tenured for 2.8 years (SD = 2.2). Work experience of participants was 4.7 years on average (SD = 5.5). Participants’ educational level was high: 40.2% finished higher vocational education and 44.7% finished university. Some participants finished intermediate vocational education (11.7%) and 3.5% indicated to have finished another type of education or none. Participants worked in the following occupational groups (ESCO classification): professionals (62%), service and sales workers (16.4%), customer services clerks and craft and related trades workers (6.2%).

The sample did not differ from sample 1 in terms of demographics but there were significant mean differences on the study variables. This sample had on average a lower score on increasing structural and social job resources (respectively, M = 3.54 versus M = 3.76, *t* = -4.73, *p* < .01, and M = 2.73 versus M = 2.98, *t* = -4.26, *p* < .01), work engagement (M = 4.44 versus 4.84, *t* = -4.92, *p* < .01), behavioral career competencies (M = 3.53 versus M = 3.77, *t* = -4.93, *p* < .01), and social support (M = 3.21 versus M = 3.51, *t* = -3.95, *p* < .01) and scored higher on autonomy (M = 3.76 versus M = 3.63, *t* = 2.13, *p* = .034). The heterogeneity of the two samples provides a good test of generalizability of the hypothesized relationships.

The results of CFAs in this sample are also presented in Table 2. To reach acceptable model fit, a correlation between two items assessing autonomy with similar content, and seven correlations within the CSES needed to be specified. Again, the latter may be due to the commonality of the items designed to measure CSE [21]. The hypothesized model fit the data significantly better than the competing model (see Table 2) and factor loadings ranged between .37 (CSE) and .94 (dedication dimension of work engagement; all *p*’s < .001).

In this sample, the composite reliabilities and average variance extracted (AVE) estimates were as follows: CSE (.81, .27), autonomy (.88, .65), social support (.82, .61), job crafting (.80, .57), career competencies (.71, .46), and work engagement (.92, .79). In line with the competing CFA, these values also suggest discriminant validity, in that the square root of the AVE for a specific construct was greater than the factor correlations between that construct and other constructs in the study [57]. This applied to all study variables except for the correlation between CSE and career competencies, which was also .69 in this sample. We retained them separately because the competing CFA indicated that they were not similar, and the correlation is not higher than .80 [58]. We also examined to what extent common method variance was present in this sample using the same analytical procedure as in sample 1. Results showed that method bias was present because the model in which the orthogonal method factor was included showed some model improvement (*χ*^2^ = 543.81, *df* = 298, Δ*χ*^2^ = 229.67/Δ*df* = 28, *p* < .01, CFI = .96, RMSEA = .05). The median amount of method variance in the 27 indicators was 13% which is lower than the results of Williams and McGonagle [61] who reported a value of 17.2% for their test of an orthogonal method factor and Williams et al.’s [62] reported median amount of method variance of 25% in prior studies.

### Hypotheses testing

Hypothesis 1 received support as the relationship between CSE and work engagement was statistically significant (*β* = .53, *p* < .01). With regard to Hypothesis 2, we found that CSE was related to autonomy (*β* = .35, *p* < .01) and social support (*β* = .28, *p* < .01). In turn, autonomy was not related to work engagement (*β* = .03, *p* = .626) but social support was (*β* = .18, *p* < .01). Hence, only social support mediated the relationship between CSE and work outcomes, providing partial support for Hypothesis 2. Using two phantom models, the results showed that the specific effect via autonomy was not significant (estimate = .02, *SE* = .04, BCC: -.044–.093, *p* = .506), whereas the specific effect via social support was significant (estimate = .11, *SE* = .04, BCC: .054–.201, *p* < .01). Thus, when only examining these specific relationships there is an indication that social support may play a role in the CSE–work engagement relationship.

**Fig 2:**
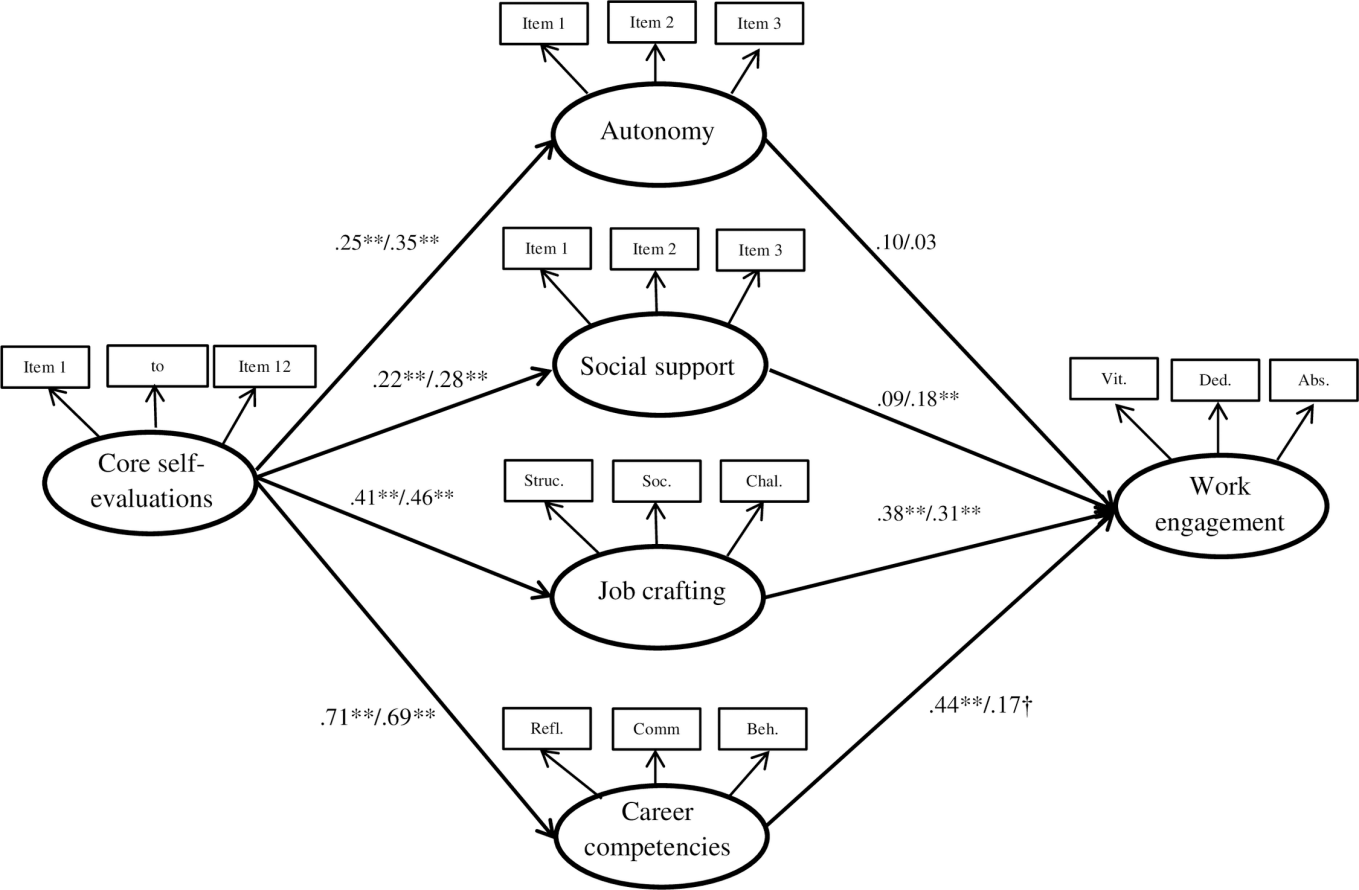
Standardized estimates of the model in which job characteristics, job crafting, and career competencies mediate the CSE–work engagement relationship. The first estimate refers to sample 1 (*N* = 303), the second estimate refers to sample 2 (*N* = 404). Struc = increasing structural job resources; Soc. = increasing social job resources; Chal. = increasing challenging job demands; Refl = reflective career competencies; Comm. = communicative career competencies; Beh. = behavioral career competencies; Vit = vigor; Ded. = dedication; Abs. = Absorption. ** *p* < .01, † *p* < .10.

In line with Hypothesis 3, CSE was significantly related to job crafting (*β* = .46, *p* < .01), and job crafting was significantly related to work engagement (*β* = .31, *p* < .01). The specific effect via job crafting estimated with a phantom model was significant (estimate: .30, *SE* = 11, BCC: .150–.506, *p* < .01). Finally, in Hypothesis 4, career competencies were examined as a mediator between CSE and work engagement. CSE was related to career competencies (*β* = .69, *p* < .01), and career competencies were marginally significantly related to work engagement (*β* = .17, *p* = .083), thus not fully supporting Hypothesis 4. The specific effect obtained for this relationship was not significant (estimate: .26, *SE* = .21, BCC: -.050–.603, *p* = .172). The direct relationship between CSE and work engagement was still present after including the mediators (from *β* = .53, *p* < .01 to *β* = .21, *p* = .009), indicating that social support and job crafting functioned as partial mediators in the relationship between CSE and work engagement (see Fig 2).

## General discussion

Although a large body of scholarly literature has shown that CSE is an important personality trait that predicts work-related outcomes, few studies have examined *why* this is the case. Our study builds upon earlier work that suggested that the positive relationship between CSE and outcomes can be explained by the perception of job characteristics [7, 23]. However, other explanations have also been proposed, namely the mediation of an action path [2], and the relevance of CSE for career development [19]. Furthermore, prior research has called for an extension of CSE findings to outcomes other than job satisfaction [4]. For these reasons, we tested three mediation processes simultaneously: a “perception path”, an “action path”, and a “development path”, and we included work engagement as the work-related outcome to capture an active well-being construct that fits the CSE construct well in terms of feeling energized and capable to reach positive work outcomes.

### Theoretical contributions

We included three mediational paths concurrently in our model. Specifically, we tested a perception path consisting of job resources, an action path consisting of job crafting, and a development path consisting of career competencies, which were all assumed to mediate the relationship between CSE and work engagement. Surprisingly, we found limited support for the perception path in our data. Whereas previous studies showed that employees high in CSEs perceived their job characteristics more positively, which in turn related to job satisfaction [46], this relationship was difficult to replicate when also taking into account the action and development path, and with work engagement as the outcome variable. Particularly autonomy, which is seen as an important job resource in job design theories [6], did not relate significantly to work engagement in either sample. However, social support did mediate the relationship between CSE and work engagement in sample 2. The finding that autonomy was not related to work engagement in both samples may imply that the action and development paths carry more explanatory power than the perception of autonomy in one’s job. One reason for this may be that job crafting and career competencies require a certain amount of autonomy, which would suggest that the perception path, at least in terms of autonomy, is inherent in both other paths. However, it can also be argued that one’s perception of the level of autonomy at work may be less strongly related to work engagement than job crafting or career competencies, which can both be considered as more “active” constructs. This notion is reinforced by our finding that job resources only played a role as mediator when not taking into account the other two paths.

Another explanation for the limited support of the perception path in this study may be related to the outcome variable in this study. Whereas previous studies have mainly focused on the relationship between CSE and job satisfaction (or job performance), we opted for work engagement for two reasons. First, by including work engagement, we were able to broaden the nomological net of CSE, as called for by Chang et al. [4]. Second, work engagement captures an individual’s enthusiasm, willingness to work, and absorption while job satisfaction captures the experience of work. It has been shown that engaged workers are hard workers because of their enthusiasm, whereas satisfied workers may be less likely to increase their effort at work because job satisfaction is passive and is based on what employees receive in return for their work versus what they expect in return from work [63]. Hence, with a more active work-related outcome, the action and development paths seem to have stronger predictive value compared to perceptions of one’s job characteristics.

Turning to the action and development path, the findings showed that employees high in CSEs are indeed more likely to proactively increase their job resources and challenging job demands, as well as develop their reflective, communicative, and behavioral career competencies. Both activities are, in turn, positively related to work engagement (though career competencies related marginally significant to work engagement in sample 2). In line with Srivastava et al. [46], we may conclude that behaviors on the job and developing career competencies can play an important role in the personality–outcome relationship. Personality is then seen as a more distal predictor of outcomes that influences how employees act and react at work. Namely, employees with a positive self-evaluation are more likely to feel capable of making changes and to feel in control of their environment and what happens to them, which coincides with actual behaviors that improve or optimize their work environment and career development such that they feel energetic and motivated.

In terms of contribution to JD-R theory [6], the present study showed that recently added mediators–personal resources [49] and job crafting [11]–play an important role in the relationship between CSE and work engagement over and above the perception of job characteristics. In addition, these findings indicate that job characteristics present in the work environment may be especially important for work-related well-being when individuals are less likely to proactively shape their work environment and develop career competencies.

Interestingly, CSE related positively to all variables in both studies. In other words, employees who regarded themselves positively also evaluated their standing on the other variables more positively. This would suggest support for the emotional generalization path [2, 7]: people’s positive feelings about themselves are likely to spill over onto their jobs. However, the current study highlights the unique explanatory power of job crafting behaviors and career competencies over and above this direct relationship and the perception of job characteristics. These findings suggest that there is more to CSE than generalization of positive self-evaluations to other outcomes. They actually enable employees to take charge in shaping their work environment and career-related competencies. Both job crafting and career competencies are important in today’s work environment in which the perception of available resources at work may decline due to the restructuring of processes [64]. Indeed, individuals who can take charge over their work and career are most likely to be successful and to experience work engagement. As our study shows, CSE may play a crucial role in achieving this.

### Research limitations

Although this study sought to build knowledge on the relationship between CSE and work outcomes by examining several mediational paths concurrently and in two separate samples, we need to recognize some limitations. First, given the cross-sectional design, causal interpretations about the specific order of the variables cannot be made. The validity of our findings is supported by the fact that we based our study on theory and previous research, and that we could largely replicate the results of sample 1 in sample 2. Yet, only longitudinal studies can uncover the directional effects among the variables. We also acknowledge that there likely is an interplay between job resources, job crafting, and career competencies (cf. JD-R Theory; [6]). For example, individuals who know what they want and know what their qualities are in work can use this knowledge to guide their job crafting actions [15]. At the same time, employees who crafted their jobs may discover what they really value in work, thereby gaining knowledge about their strengths and motives (i.e., career competencies). Future research should elaborate on the potential reciprocity of these relationships.

Another limitation concerns the use of self-reports to assess the study variables. Although statistical tests and the results do not give rise to serious concerns in the data–variables could be distinguished from each other, correlations are not suspiciously high, and a conservative test for CMV indicated little concern–future studies could use a design with other-ratings or objectively assessed outcomes as well. Despite the limitations, the use of two samples from different countries serves as a cross-validation of our findings, providing additional support for most parts of the hypothesized model. Moreover, the study presents a promising first empirical test of the theorized paths that should encourage interested researchers to continue this line of research with more advanced research methods.

### Practical implications

Given that CSE is a personality trait, it may be valuable to assess job candidates on their level of CSE and select those who have a positive self-view to fill vacancies within the organization. Other personality traits, such as the Big Five, are already often used in assessment procedures, and our study suggests that it would be valuable to add CSE to such assessments. However, this would be only be possible when selecting new employees. Employers can also increase the likelihood of having engaged employees by reflecting on how they develop and shape jobs. Namely, when jobs are characterized by job rotations that allow employees to accumulate new work experiences [38], or that allow for proactive job crafting and the development of career competencies, employees are most likely to experience high work engagement [11, 14]. Job crafting and career competencies have also been found to be trainable [47, 65], implying that they can be both directly and indirectly stimulated by managers or organizational policies.

### Conclusion

CSE is an important predictor of organizationally relevant outcomes. Although knowledge about the relevance of CSE is accumulating, the present study contributed to the “why” question of the role of CSE in predicting work engagement by incorporating several mediational paths in the same study using two separate samples. We found that CSE related to job characteristics (perception path), job crafting (action path) and career competencies (development path). In addition, we found that social support, job crafting and career competencies functioned as (partial) mediators in the relationship between CSE and work engagement. CSE thus not only influences job perceptions but also prepares individuals to be active at work and in their career development, which is related to a positive experience of work.

## Supporting information

S1 File Glossary of terms. (DOCX)

S2 File Dataset. (SAV)
