# Supplementary material for: Core self-evaluations and work engagement: Testing a perception, action, and development path
Source: PLoS One. 2017 Aug 7;12(8):e0182745. doi: 10.1371/journal.pone.0182745 (PMC5546708; doi:10.1371/journal.pone.0182745)
Supplement: S1 File — (DOCX) [file pone.0182745.s001.docx]

**Glossary of Terms**

**Approach and avoidance motivation:**

Approach motivation is guided by the idea that behavior is driven by or directed toward positive stimuli. This means that individuals with an approach motivation behave in such a way that they promote the experience of new positive stimuli and try to maintain existing positive stimuli. These positive stimuli can represent a range of outcomes such as objects, events, or possibilities [5]. Avoidance motivation refers to the opposite: individuals with an avoidance motivation are inclined to pursue goals that are related to preventing or avoiding negative experiences [8].

**Autonomy:**

Autonomy is a job resource that refers to the level of freedom one has at work in terms of scheduling and/or performing work activities [53]. Autonomy allows one to certain make choices and decisions about one’s own work tasks and procedures [28].

**Career competencies:**

Career competencies refer to the knowledge, skills, and abilities central to career development [17], such as reflecting on one’s motivation and identity, skills and expertise, and relationships and reputation [18]. Career competencies consist of three interrelated competencies [17]: reflective, communicative, and behavioral career competencies. Reflective career competencies refer to a person’s reflection on career motivation and qualities. Communicative career competencies deal with networking and self-profiling. Finally, behavioral career competencies refer to work exploration and career control.

**Core self-evaluations (CSE)**:

CSE is broad measure of one’s self-perceptions and is defined by four higher-order concepts, namely self-esteem, generalized self-efficacy, locus of control, and emotional stability [23]. Self-esteem refers to how people evaluate their own self-worth, and generalized self-efficacy captures whether individuals trust to have the ability to perform and cope successfully across a range of life situations [1]. Locus of control refers to one’s belief to be able to impact the environment to reach desired outcomes, and finally, emotional stability reflects the disposition to feel calm and secure and a sensitivity to positive emotional states [8].

**Job crafting:**

Job crafting refers to self-initiated behaviors aimed to make changes in job resources and job demands such that the job better fits their preferences, skills, and abilities [11]. Employees craft their job resources by increasing them because of their motivating potential [34]. Furthermore, job demands can be crafted either by decreasing those aspects that hinder achieving work goals, or by increasing those job demands that are considered challenging though still require effort [35]. Four job crafting dimensions can be distinguished [11]: increasing structural job resources (e.g., mobilizing autonomy and variety), increasing social job resources (e.g., mobilizing social support and feedback), increasing challenging job demands (e.g., mobilizing working on new projects), and decreasing hindering job demands (e.g., decreasing cognitive job demands).

**Job demands-resources theory (JD-R):**

According to the JD-R theory [34], the characteristics of work environments can be distinguished in two general categories that are referred to as job demands and job resources. The basic assumption is that each work environment is characterized by its own specific job demands and job resources. In general, job demands are defined as those physical, social, or organizational aspects of the job that require physical and/or psychological effort from employees. As a consequence, job demands are related to physiological and/or psychological costs particularly when individuals experience heavy job demands. Job resources refer to work aspects that enable employees to do their work (e.g., freedom to decide how to do work), that can buffer the effects of certain stressors (e.g., social support when something stressful happened) and work aspects that can help employees to learn new things (e.g., opportunities for development) [27].

The JD-R model further specifies that job demands may deplete employees’ resources and lead to strain while job resources lead to motivation. A final premise of the JD-R model is that job resources can weaken the relationship between job demands and strain such that dealing with these demands is manageable due to the availability of job resources.

**Job satisfaction:**

Job satisfaction refers to a pleasurable emotional state that results as a function of the perceived relationship between what one wants from a job and what one perceives it as offering or entailing [66].

**Personal resources:**

Personal resources refer to a sense of being able to control and impact the environment successfully [49]. These resources originate from within the self and are generally linked to the ability to deal with all kinds of circumstances. Examples of personal resources are hope and optimism.

**Supervisor support:**

Supervisor support reflects the extent to which employees have the feeling that their supervisor provides encouragement and support to them.

**Work engagement:**

Work engagement is defined as a “positive work-related state of fulfillment that is characterized by vigor, dedication, and absorption” [25, p. 701]. Vigor refers to high levels of energy, dedication refers to enthusiasm and involvement in one’s work, and absorption arises when one is fully concentrated at work in such a way that it is difficult to detach oneself from work [25].
